# A retrospective analysis of survival prognostic factors and risk stratification in recurrent glioblastoma

**DOI:** 10.1080/07853890.2025.2533435

**Published:** 2025-09-24

**Authors:** Lei She, Han Wu, Xin Zhang, Chunmao Zheng, Lin Su

**Affiliations:** a Department of Oncology, Xiangya Hospital, Central South University, Changsha, China; b Department of Cardiothoracic Surgery, The First People’s Hospital of Chenzhou, Xiangnan University, Chenzhou, China; c Department of Pharmacy, The First People’s Hospital of Chenzhou, Xiangnan University, Chenzhou, China; d National Clinical Research Center for Geriatric Disorders, Xiangya Hospital, Central South University, Changsha, China

**Keywords:** Nomogram, prediction, recurrent glioblastoma, risk stratification, survival

## Abstract

**Background:**

Traditional Cox analysis identifies independent prognostic factors in recurrent glioblastoma (rGBM) but often overlooks their interrelationships. We aimed to develop a predictive nomogram integrating these multifaceted factors to establish a clinically applicable risk stratification model.

**Materials and methods:**

In a retrospective analysis of IDH-wildtype rGBM, we used Cox regression to evaluate prognostic factors including age, sex, Ki-67 index, Karnofsky Performance Status (KPS), time to first progression, number of recurrent lesions, tumor location, O^6^-methylguanine-DNA methyltransferase (MGMT) methylation status, and post-recurrence treatment. Significant predictors were used to construct a nomogram in R software, generating a risk stratification model by converting risk scores into categorical levels. The model underwent bootstrap validation.

**Results:**

Our cohort included 206 patients with a median overall survival (OS) of 8.3 (95% CI, 7.2–9.4) months. Multivariate analysis revealed KPS > 50 (*p* = 0.009; HR 0.61, 95% CI: 0.42–0.88), MGMT methylation (*p* = 0.033; HR 0.68, 95% CI: 0.48–0.97), time to first recurrence >12 months (*p* = 0.048; HR 0.69, 95% CI: 0.47–1), single lesion (*p* = 0.005; HR 0.63, 95% CI: 0.46–0.87), and post-recurrence therapy (surgery: HR 0.35, 95% CI: 0.21–0.59; targeted therapy/Tumor-treating fields (TTF)/re-irradiation: HR 0.5, 95% CI: 0.35–0.71; both *p* < 0.001) were favorable independent prognostic factors for OS. The nomogram-based risk stratification model successfully stratified patients into low-, medium-, and high-risk groups, yielding distinct OS outcomes (13.9 vs. 6.5 vs. 3.98 months; *p* < 0.0001). Its predictive performance was confirmed with area under curve (AUC)s of 0.76, 0.72, and 0.73 at 6, 12, and 24 months, respectively.

**Conclusions:**

We developed and internally validated a nomogram-based risk stratification model for rGBM. By integrating key clinical and molecular factors, this tool accurately predicts patient survival.

## 1. Introduction

Among primary brain tumors, glioblastoma (GBM) is the most common and aggressive [1,2]. All patients inevitably experience recurrence despite standard treatment (maximum surgical resection with the assurance of safety combined with concurrent chemoradiotherapy and adjuvant temozolomide (TMZ) chemotherapy) [3,4]. Currently, there is no uniform treatment for recurrent GBM (rGBM). Among chemotherapeutic agents, nitrosoureas such as lomustine have limited efficacy [5,6]; the efficacy of TMZ has not been consistent across clinical studies [7,8]. Among targeted agents, bevacizumab does not appear to prolong overall survival (OS) in rGBM patients [9]. The tyrosine kinase inhibitor (TKI) regorafenib has shown an encouraging survival advantage in the treatment of rGBM [10]. Anlotinib in China may also be a new potential therapeutic agent [11]. Tumor-treating fields (TTF) has not shown a survival advantage in rGBM [12]. Re-irradiation may control local disease in some patients, but the cumulative risk of neurotoxicity remains worrisome[13,14]. If appropriate, reoperation is also an option, and patients with rGBM may benefit from reoperation [15,16]. In addition, some studies have shown longer survival in patients receiving combination therapy following recurrence [17,18].

Apart from salvage treatment strategies, several factors—including patient age, Karnofsky Performance Status (KPS), O6-methylguanine-DNA methyltransferase (MGMT) promoter methylation status, Ki-67 labeling index, and progression-free survival (PFS; the interval between initial therapy and recurrence)—are critical determinants of prognosis in patients with recurrent glioblastoma [19]. Evidence from prior literature demonstrates that younger patients typically achieve superior survival outcomes [20]. Higher KPS scores are linked to improved prognosis and greater tolerability of salvage therapies [21]. MGMT promoter methylation is associated with enhanced responsiveness to alkylating agents, particularly temozolomide [22]. Conversely, a higher Ki-67 labeling index correlates with increased tumor aggressiveness and diminished survival rates [23]. Notably, a longer PFS prior to recurrence usually suggests a more indolent tumor biology and potentially greater responsiveness to subsequent interventions [24]. In view of the complex interplay among these variables, individualized therapeutic strategies that integrate patient-specific genetic and clinical features are essential for optimizing outcomes in patients with rGBM.

Therefore, understanding the complex interplay among clinical, molecular, and therapeutic factors is pivotal for enhancing clinical outcomes and guiding therapeutic decisions in patients with recurrent glioblastoma. Although traditional Cox multivariate analysis can identify independent prognostic factors, it often fails to elucidate the intricate interrelationships among them. This limitation prompted our hypothesis that a predictive nomogram integrating these multifaceted factors could be established, thereby providing the foundation for a clinically applicable risk stratification model.

## 2. Methods

### 2.1. Case data

Data were collected retrospectively from Xiangya Hospital of Central South University between December 2011 and June 2022. The main inclusion criteria included: (1) GBM (IDH wild-type) in the initial histopathological specimen according to the 2021 WHO Classification of Tumors of the Central Nervous System [25]; (2) concurrent chemoradiotherapy and adjuvant chemotherapy after surgical resection, and (3) regular head magnetic resonance imaging (MRI) examinations, and tumor progression according to RANO criteria. (4) Complete follow-up data after tumor progression. At the same time, clinical variables such as gender, age, Ki-67 labeling index, KPS at recurrence, time from the initial diagnosis to first recurrence, site of recurrence, mode of recurrence, and post-recurrence treatment regimen were also collected to explore their relationship with survival. The selection of clinical variables for this study was guided by evidence from existing literature and their established roles as prognostic factors in oncology. We aimed to include a comprehensive set of predictors covering multiple domains. Throughout the retrospective data collection process, particular attention was paid to ensuring consistency between radiological and pathological reviews, thereby rigorously maintaining data quality and integrity. As this was a retrospective study, the Medical Ethics Committee of Xiangya Hospital of Central South University granted exemption from written informed consent (Ethics No. 2024111523). To address potential ethical concerns, all patient data were anonymized prior to analysis, and no personally identifiable information was included in the dataset. Access to raw data was strictly limited to authorized research staff in accordance with institutional and national regulations, thereby ensuring full protection of patient confidentiality and data security.

### 2.2. Statistical analysis

The primary endpoint of the study was OS in rGBM, which was measured as the time between the first progression of tumors and death from any cause. For statistical analysis, SPSS 25.0 and R 4.2.2 software were used. Patients’ baseline data were analyzed by direct counting method, and measurement data were expressed as mean (standard deviation). Univariate and multivariate analyses related to survival used Cox proportional hazards models, and variables with *p* < 0.1 in univariate analysis were considered in the multivariate analysis. To predict the OS rate at 6 months, 1 year and 2 years, a nomogram model was developed using R software based on the results of univariate and multivariate analyses. The bootstrap method was used to perform 1000 samplings with the equal number of releases to validate the nomogram model. The discrimination, accuracy, and clinical applicability of the model were tested using receiver operating characteristic (ROC) curves, calibration curves, and decision curve analyses.

### 2.3. From nomogram to risk stratification model development

To facilitate clinical use, the risk degree of prognostic factors in a nomogram were generated from corresponding integers. Each patient received a total risk score. The optimal risk stratification cut-off value was obtained using the X-tile (version 3.6.1) software [26]. In order to compare survival rates between various risk groups, Kaplan–Meier survival curves were used and log-rank tests were performed to measure differences between groups. *p* < 0.05 indicates a significant difference. The risk stratification model was also validated using the Bootstrap technique. It was also required to assess the clinical utility, calibration, and discriminating power of the risk stratification model.

## 3. Results

### 3.1. Patient characteristics

**Figure 1.**
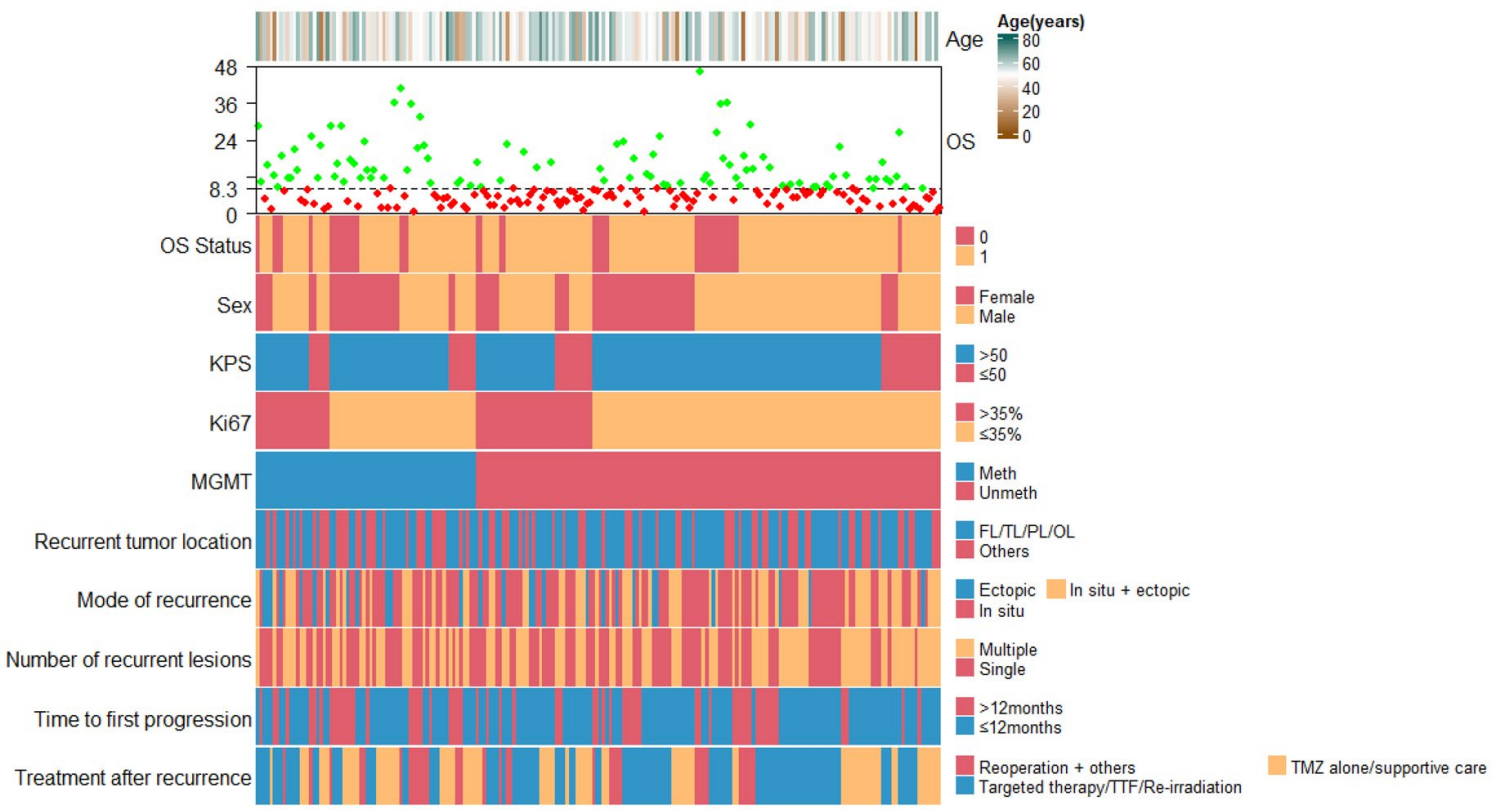
Schematic summary of clinic characteristics in rGBM. rGBM, recurrent glioblastoma; OS, overall survival; KPS, Karnofsky performance status; MGMT, O6-methylguanine-DNA methyltransferase; meth, methylation; unmeth, unmethylated; FL, frontal lobe; TL, temporal lobe; PL, parietal lobe; OL, occipital lobe; RT, radiotherapy; TMZ, temozolomide; TTF, tumor-treating fields.

206 patients with rGBM were included in the study. There was a median age of 50 years. There were 129 males and 77 females. There were 163 patients with KPS >50 and 43 patients with KPS ≤ 50. 57 patients had Ki-67 labeling index >35% and 149 had ≤35%. MGMT was methylated in 66 individuals and unmethylated in 140. At recurrence, 102 patients had single tumors and 104 patients had multiple tumors. After recurrence, 73 patients were treated with TMZ alone or supportive care, 101 patients were treated with targeted therapy, TTF or re-irradiation, and 32 patients underwent reoperation combined with other treatments. Table 1 and Figure 1 shows the detailed characteristics of patients.

**Table 1.** Clinical data and characteristics of rGBM patients (*n* = 206).

Characteristic	No. of patients (%)
Age (years)	
Mean ± SD	50.0 ± 12.5
Sex	
Male	129 (62.6%)
Female	77 (37.4%)
Ki**-**67 labeling index (%)	
≤35	149 (72.3%)
>35	57 (27.7%)
KPS	
≤50	43 (20.9%)
>50	163 (79.1%)
Time to first progression (months)	
≤12	145 (70.4%)
>12	61 (29.6%)
Number of recurrent lesions	
Single	102 (49.5%)
Multiple	104 (50.5%)
Mode of recurrence	
*In situ*	111 (53.9%)
Ectopic	27 (13.1%)
*In situ* + ectopic	68 (33.0%)
Recurrent tumor location	
FL/TL/PL/OL	129 (62.6%)
Others	77 (37.4%)
MGMT	
Meth	66 (32.0%)
Unmeth	140 (68.0%)
Treatment after recurrence	
TMZ alone/supportive care	73 (35.4%)
Targeted therapy/TTF/re-irradiation	101 (49.0%)
Reoperation + others	32 (15.5%)

rGBM, recurrent glioblastoma; SD, standard deviation; KPS, Karnofsky performance status; MGMT, O6-methylguanine-DNA methyltransferase; Meth, Methylation; Unmeth, Unmethylated; FL, Frontal lobe; TL, Temporal lobe; PL, Parietal lobe; OL, Occipital lobe; RT, Radiotherapy; TMZ, Temozolomide; TTF, Tumor-treating fields.

### 3.2. Prognostic factor analyses

**Figure 2.**
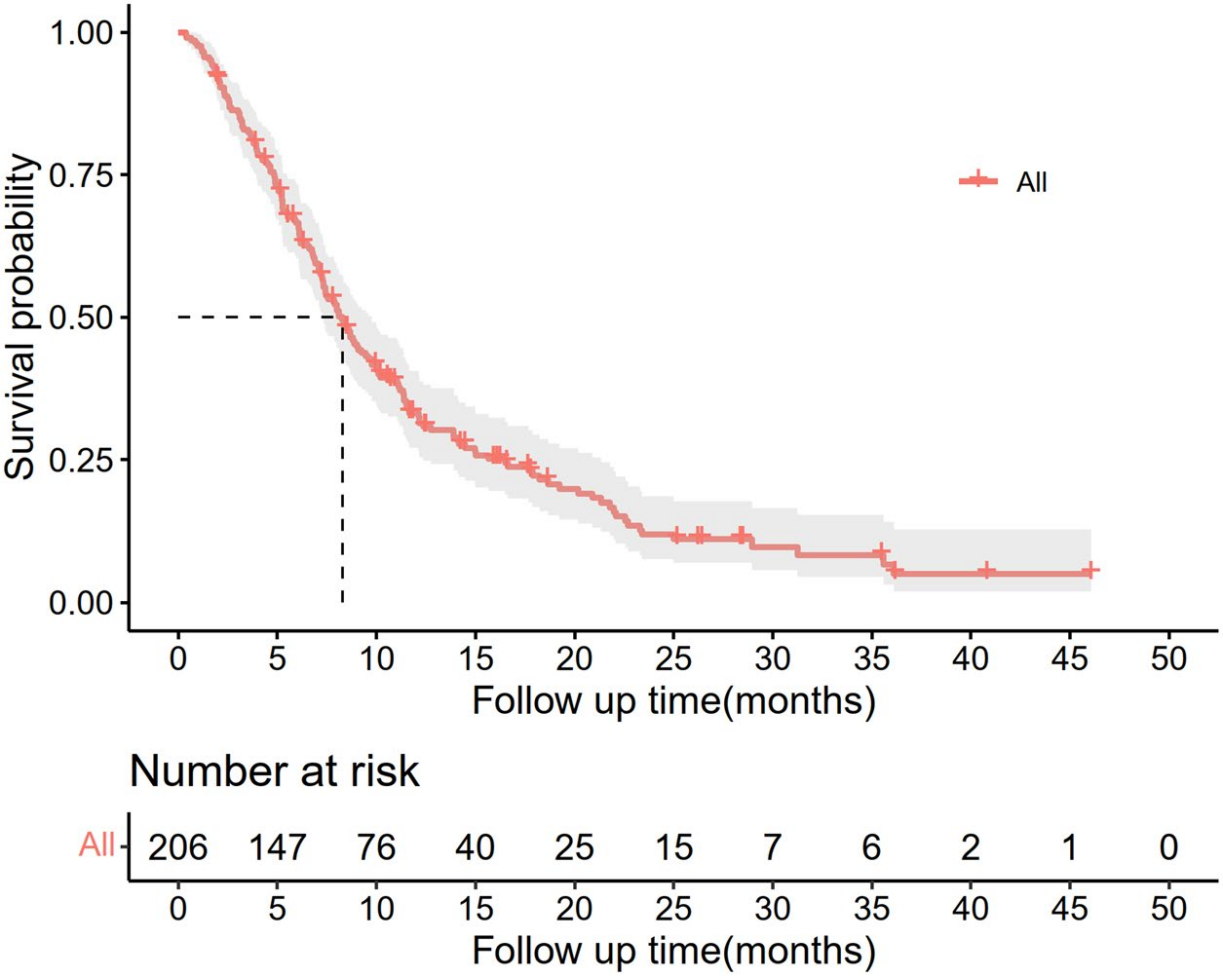
Kaplan–Meier curves estimating overall survival of patients with recurrent glioblastoma.

The median follow-up period was 26.4 months (95% CI: 22.5–30.4). During follow-up, 166 (80.6%) patients died. Median OS was 8.3 (95% CI: 7.2–9.4) months. There was a 66.5% OS rate at 6 months, 33.1% at 1 year, and 11.9% at 2 years (Figure 2).

**Figure 3.**
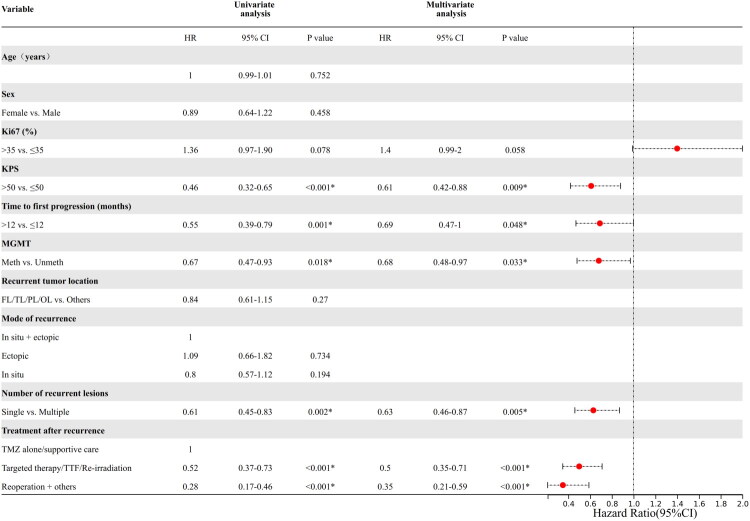
Univariate and multivariate cox regression analysis of overall survival in recurrent glioblastoma. KPS, Karnofsky performance status; MGMT, O6-methylguanine-DNA methyltransferase; meth, methylation; unmeth, unmethylated; FL, frontal lobe; TL, temporal lobe; PL, parietal lobe; OL, occipital lobe; TMZ, temozolomide; TTF, tumor-treating fields.

Patients with KPS >50 (*p* < 0.001), MGMT methylation (*p* = 0.018), time to first recurrence >12 months (*p* = 0.001), single lesions at recurrence (*p* = 0.002), reoperation after recurrence (*p* < 0.001), and targeted therapy/TTF/re-irradiation after recurrence (*p* < 0.001) were found to have better OS by univariate analysis. By multivariate analysis, KPS >50 (*p* = 0.009; HR 0.61, 95% CI 0.42 − 0.88), MGMT methylation (*p* = 0.033; HR 0.68, 95% CI 0.48 − 0.97), time to first recurrence >12 months (*p* = 0.048; HR 0.69, 95% CI 0.47 − 1), single lesions at recurrence (*p* = 0.005; HR 0.63, 95% CI 0.46 − 0.87), reoperation after recurrence (*p* < 0.001; HR 0.35, 95% CI 0.21 − 0.59), and targeted irradiation/TTF/re-irradiation after recurrence (*p* < 0.001; HR 0.5, 95% CI 0.35 − 0.71) were found to be independent prognostic factors for favorable OS (Figure 3).

### 3.3. Nomogram model evaluation and verification

**Figure 4.**
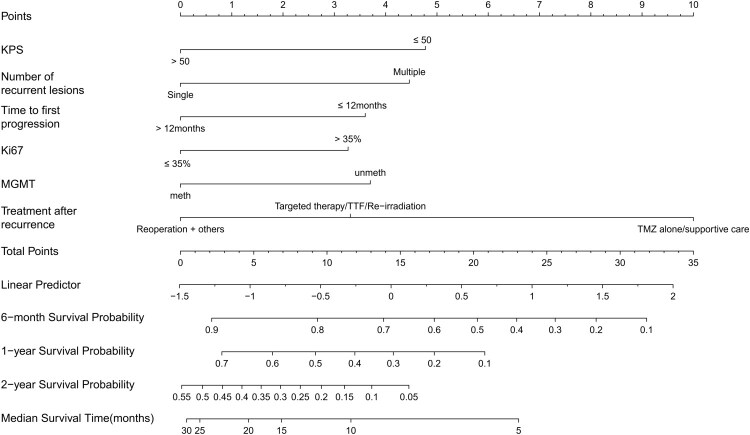
Nomogram model for predicting the overall survival of recurrent glioblastoma. KPS, karnofsky performance status; MGMT, O6-methylguanine-DNA methyltransferase; meth, methylation; unmeth, unmethylated; FL, frontal lobe; TL, temporal lobe; PL, parietal lobe; OL, occipital lobe; TMZ, temozolomide; TTF, tumor-treating fields.

**Figure 5.**
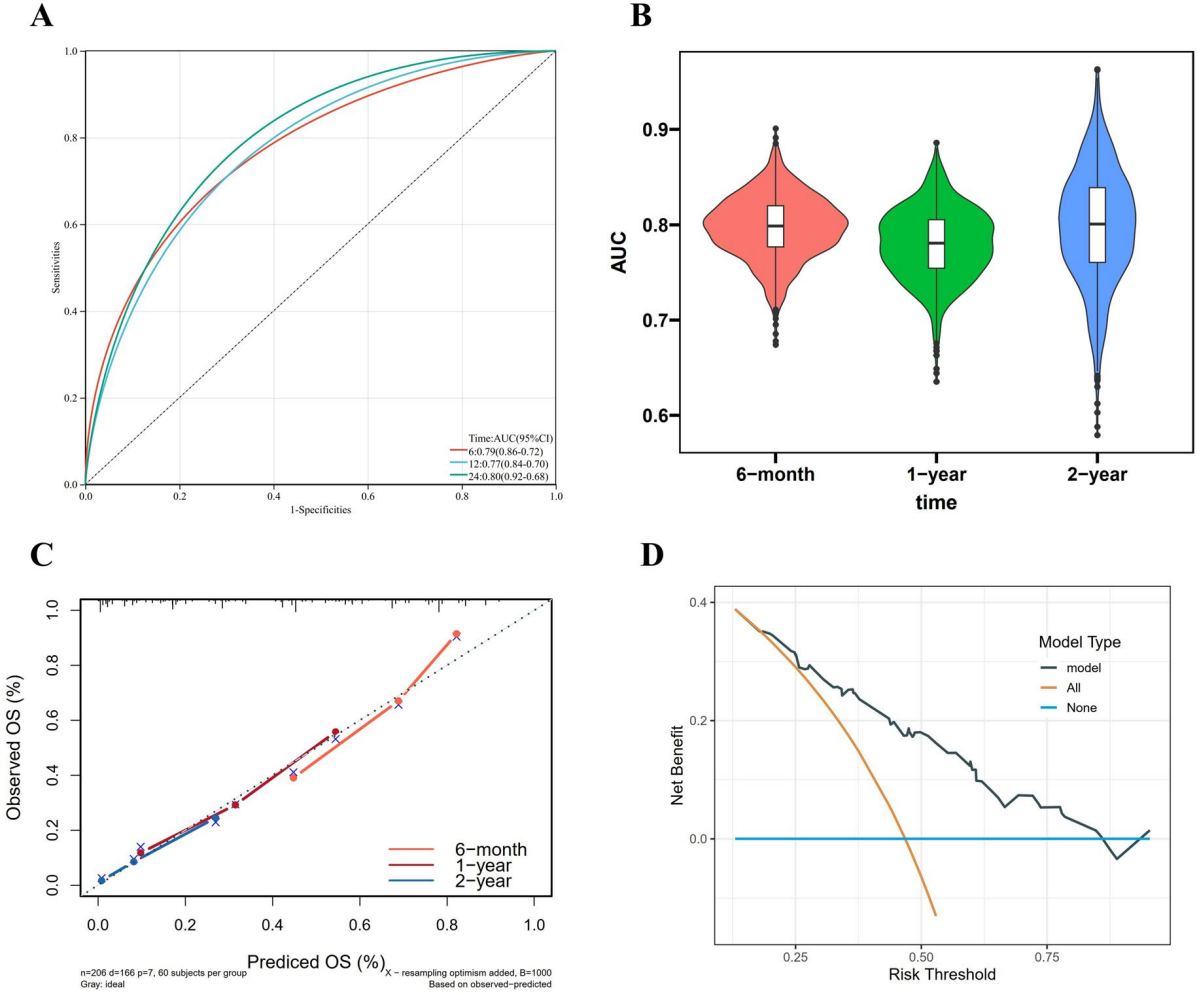
The ROC curve of nomogram model for OS(a). AUC range of bootstrap samples of the nomogram (B). Calibration curves of the nomogram (C). DCA of the nomogram (D). OS, overall survival; ROC, receiver operating characteristic; AUC, area under curve; DCA, decision curve analysis.

The 6-month, 1-year and 2-year survival of patients with rGBM were predicted using a nomogram with predictive variables (Figure 4). There were 0.79 (95% CI: 0.72–0.86), 0.77 (95% CI: 0.70–0.84), and 0.80 (95% CI: 0.68–0.92), respectively, for area under curve (AUC)s at 6 months, 1 year, and 2 years (Figure 5A). The ROC curve over time revealed an AUC above 0.7. Following bootstrap resampling, the mean AUC values at 6 months, 1 year, and 2 years were 0.8, 0.78, and 0.80, respectively (Figure 5B). This suggests that the model has good discrimination. A satisfactory agreement was found between the actual and predicted OS probabilities in the calibration curve (Figure 5C). In addition, decision curve analysis (DCA) showed considerable clinical applicability of the model (Figure 5D).

### 3.4. Risk stratification model evaluation and verification

**Figure 6.**
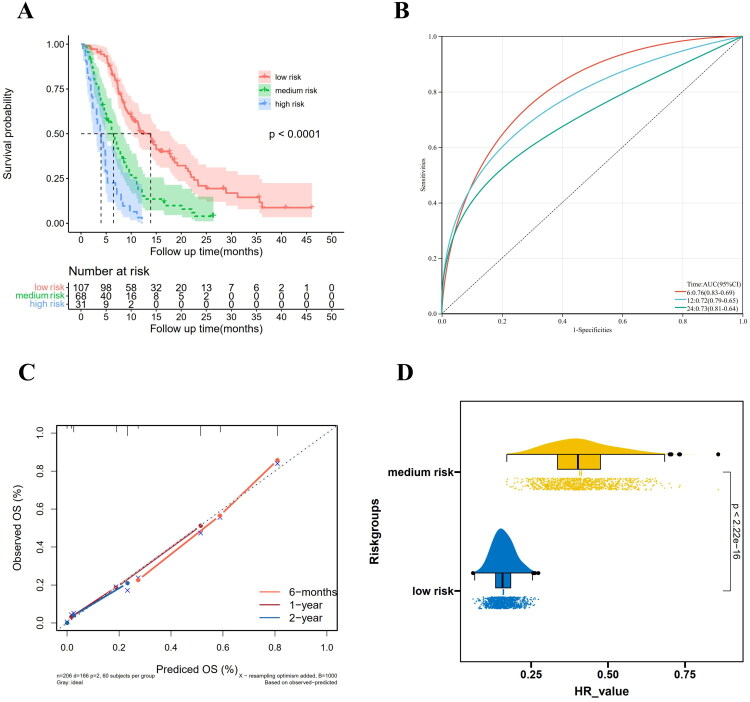
OS curve of three risk groups (a). The ROC curve of risk stratification model for OS(B). Calibration curves of the risk stratification model (C). HR range of bootstrap samples of the risk stratification model (D). OS, overall survival; ROC, receiver operating characteristic; AUC, area under curve; HR, hazard ratios.

In order to make it easier for physicians to utilize this predictive model in their clinical practice, we modified each prognostic factor of the nomogram to a scoring integer: KPS ≤50 (5 points), multiple recurrence sites (4 points), time to first progression ≤12 months (4 points), Ki-67 labeling index >35% (3 points), MGMT unmeth (4 points), targeted therapy/TTF/re-irradiation (3 points), TMZ alone/supportive care (10 points) (Table 2). In this way, each patient received a total risk score. We used X-tile software to obtain optimal cutoffs of 14 and 21, and then divided patients into high, medium, and low-risk groups of >21, 15–21, and ≤14. Median OS was 3.98 (95% CI: 2.33–5.22) months, 6.5 (95% CI: 5.06–8.34) months, and 13.9 (95% CI: 10.2–17.87) months in the 3 groups, respectively. Survival was significantly different among the 3 groups (*p* < 0.0001) (Figure 6A). Medium-risk patients had an HR of 0.41 (95% CI 0.26–0.64; *p* < 0.001) compared to high-risk patients. Low-risk patients had an HR of 0.16 (95% CI 0.1–0.26; *p* < 0.001). Survival rates at 6 months, 1 year, and 2 years for patients in each risk group were detailed in Table 3. AUC at 6 months, 1 year, and 2 years were 0.76 (95% CI: 0.69–0.83), 0.72 (95% CI: 0.65–0.79), and 0.73 (95% CI: 0.64–0.81), respectively (Figure 6B). The calibration curve was satisfactory (Figure 6C). The survival of the three groups was significantly different, with a median *P* value of 2.87E–18 (interquartile range: 5.69E–21–1.17E–15). Compared to high-risk patients, the median HR was 0.40 (interquartile range: 0.34–0.48) for the medium-risk group and 0.16 (interquartile range: 0.13–0.18) for the low-risk group. (Figure 6D).

**Table 2.** Prognostic factor score developed from a nomogram.

Prognostic factor	Score generated from nomogram (points)	Score rounded from nomogram (points)
KPS ≤50	4.77	5
Multiple recurrence sites	4.46	4
Time to first progression ≤12months	3.6	4
Ki-67 labeling index >35%	3.27	3
MGMT unmethylated	3.7	4
Targeted therapy/TTF/Re-irradiation	3.31	3
TMZ alone/supportive care	10	10

KPS, Karnofsky performance status; MGMT, O6-methylguanine-DNA methyltransferase; TMZ, Temozolomide; TTF, Tumor-treating fields.

**Table 3.** Overall survival rate of patients with risk stratification.

Risk stratification	HR	95% CI	*p* value	6-month OS rate	1-year OS rate	2-year OS rate
High risk	1	/	/	22.6%	3.2%	0
Medium risk	0.41	0.26–0.64	<0.001	56.5%	18.6%	3.9%
Low risk	0.16	0.1–0.26	<0.001	85.8%	51.2%	20.9%

HR, Hazard ratio; CI, Confidence interval; OS, Overall survival.

## 4. Discussion

Because GBM is highly invasive and malignant, the lesions are mostly located in important intracranial functional areas, and the prognosis of patients is poor. Despite standard treatment, nearly all GBM patients experience tumor recurrence. rGBM has a very poor prognosis and there are currently no standardized treatment decisions. In this real-world study, we explored the most valuable prognostic indicators for rGBM patients and then developed a predictive model.

In our study, from the time of recurrence, the median OS of the patients was 8.3 months. OS rates were 66.5%, 33.1%, and 11.9% at 6 months, 1 year, and 2 years. Survival results were similar to or slightly better than previously published studies [20,24].

Among the many factors associated with prognosis in rGBM patients, we found that treatment regimens after progression were the strongest prognostic factors affecting OS. Targeted therapy/TTF/re-irradiation patients had a lower risk of death (HR 0.5) compared with supportive care or TMZ alone, whereas patients who underwent reoperation had a significantly lower risk of death (HR 0.35). Stavrinou et al. collected data from two centers in Greece and Germany to analyze the treatment patterns of GBM patients after recurrence and found that patients with multimodal second-line treatment survived significantly better than patients with supportive care or only one therapeutic modality [17]. Archavlis et al. included 90 patients with rGBM and showed that patients treated with the combined scheme of salvage treatments had longer survival compared with TMZ alone (5 months vs 8 months, *p* = 0.043) [18]. A meta-analysis by Zhao included 21 articles (8630 patients) and showed that reoperation was associated with a longer OS when considered as a fixed covariate (HR = 0.66, 95% CI: 0.61–0.71, *p* < 0.001) [27]. Another multicenter retrospective study included 503 rGBM patients at 20 institutions to investigate the clinical benefit of re-resection in rGBM patients [16]. Conclusions showed that reoperative resection may contribute to prolonged survival in rGBM patients under the premise of an acceptable complication rate. There are also some findings that support the efficacy of combination therapy in patients [28–30].

KPS has been considered an important prognostic variable in rGBM patients. High KPS patients are more likely to receive aggressive treatment, which is an important factor. Audureau et al. showed that patients with KPS ≤80 at recurrence had a worse prognosis [20]. Park et al. also showed that KPS ≤80 was associated with poor prognosis in rGBM patients (*p* = 0.03) [21]. In two other studies, performance score (PS) was identified as a major prognostic factor for OS in rGBM patients [24,31]. While our study found that patients with KPS >50 at progression had better OS, the multivariate analysis also identified KPS >50 as a favorable independent prognostic factor for OS. Although KPS in our study did not have the same threshold as other studies, it remains considered an objective survival indicator in rGBM patients.

According to the study that only included IDH wild-type rGBM patients, time from the initial diagnosis to first recurrence was an independent prognostic factor for OS; patients with PFS >11 months had better OS after recurrence (*p* = 0.02) [32]. Multivariate analysis in another study showed that the recurrence-free interval is also an independent prognostic factor for OS in patients with rGBM (*p* = 0.048) [24]. Similarly, our study showed patients with time to first recurrence >12 months had a better prognosis.

Our study also found patients with single lesions at recurrence had a better prognosis than multiple lesions, which was also similar to previous findings [24,31]. The possible reason is that multiple recurrent lesions invade more functional sites and thus cause increased damage to the body. In addition, the DIRECTOR study showed that rGBM patients with MGMT promoter methylation could still benefit from TMZ chemotherapy after completion of standard therapy [33]. In our study, patients with MGMT methylation had a better prognosis, possibly in part due to the use of TMZ in combination therapy.

This was currently one of the few retrospective studies that included only IDH wild-type rGBM, and all patients underwent standard first-line therapy before recurrence. We constructed a nomogram model based on the results of COX regression analysis to determine prognostic factors for OS after recurrence. It was a novel approach to generating a risk stratification model based on a nomogram. We observed a small change in AUC between the nomogram and the newly developed risk stratification model. This new quantitative tool showed good performance in bootstrap method validation. A distinguishing feature of our proposed risk stratification model is its dual capacity to accurately predict patient survival while simultaneously offering exceptional clinical utility. The model’s strength is anchored in its reliance on readily accessible variables—encompassing clinical characteristics, standard radiological findings, and routine pathological assessments. This deliberate inclusion of commonly available parameters ensures its broad applicability beyond specialized academic centers, facilitating widespread clinical adoption. Consequently, our risk stratification model represents not merely an alternative to existing prognostic tools but a substantial and practical improvement. It provides clinicians with a more accurate, generalizable, and user-friendly instrument to inform the development of tailored therapeutic strategies for patients with rGBM.

Obviously, this study has some limitations as well. First, the model building for this study is based on retrospective data and lacks more detailed molecular and imaging data, so there remains room for progress in power of the model. Perhaps a more accurate OS model could be obtained by adding prognostic genomic features or relevant biomarkers. In addition, nearly all data from our center for nearly 10 years were included in this study. Although repeated sampling verification worked well, unfortunately, there was no external verification. Finally, the treatment options for patients after relapse were numerous and complex, some of which were heterogeneous. Basically, we divided the treatment options into 3 categories in general, without subdivision. In fact, different treatment options can also affect the prognosis of patients. Therefore, there is an urgent need for multi-site validation of this model in more populations to provide high-level evidence for its future in clinical applications. It is also hoped that additional potentially relevant prognostic factors will be added to improve the construction of the model.

## 5. Conclusion

In conclusion, our study found that MGMT methylation status, Ki**-**67 labeling index, KPS, time to first recurrence, number of recurrent target lesions, and treatment regimen after recurrence were significant factors affecting OS. A risk stratification model based on a nomogram can provide a prognostic reference for rGBM patients. This model can help clinicians develop optimal treatment strategies. Of course, it needs to be further validated in external multi-center studies.

## Data Availability

The data presented in this study are available on request from the corresponding author. The data are not publicly available due to privacy.
